# Monthly Summary

**Published:** 1898-04

**Authors:** 


### Monthly Summary.

HINTS.—BY A LAZY MAN.—1. You can cut vulcanite with a file better when both are dry than wet. If you think you need a new file, dip your plate in dry plaster of paris, rub it off with a wheel-brush, and ofter you will find that the fault is not in the file.

2. After you have polished your plate, sprinkle a little dry plaster on it, and give it a run over the soft wheel-brush.

3. Tack a square piece of bed-tick or apron cloth under the part of the bench where you work. It will always be there. Pull it over your knees when you sit there to work. Keeps your pantaloons clean, and often keeps you from saying naughty words, as it catches teeth you might drop on the floor.

4. When your rubber overshoes wear out, keep the soles. Very handy if fastened on a block convenient to use. The heels make good bench blocks.

5. Keep your old kid gloves and wear them when sand-papering or polishing artificial sets. Rub vaseline into your finger-nails, and over your fingers before putting on the gloves.

6. When your dental engine kicks or makes a noise at the joints, oil it a little. If that does not stop the row, do not swear at the manufacturer until you have got down on your marrow-bones and tightened up screws that may be loose. If that succeeds shake hands with yourself; if it does not, smash something. It will ease your mind.

7. Cover your dental engine with a cover the shape of an old candle extinguisher.—*Dominion Dental Journal.*

---

PRESCRIPTION WRITING.—BY L. D. S.—The *New York Medical Journal* quotes from an article in a druggist's periodical, on the subject of drug names and prescription writing, which may apply in part to dental practice. The prescription pad is not used as much as it should be by the dentist, and in the matter of lotions, etc., for special pathological condition of the oral cavity within the prerogative of our practice, we have many important demands upon our knowledge of prescribing. While we run no such risks as the general physician, even our limitations demand great care to avoid confusion and error, not to speak of the less formidable mistakes of chemical incompatibility. The *New York Medical Journal* quotes the following formula.

"℞ Hydr. chlor. - - - - gr. xx.

Tinct. hyosey. - - - - ʒ i.

Aqæ. - - - - - - ʒ ix.

"M. Draught to be taken at bed time."

"Which chloride of mercury is intended?" asks the writer. "The context shows plainly that neither is wanted but chloral hydrads. Is it not all wrong that human life should thus be trifled with?"

To which we may add, "Does it not seem all wrong that the laws of a State permit the most dangerous prescriptions to be compounded by young men of meagre and insufficient preliminary and practical training?" We happen to be familiar with the requirements for the practice of pharmacy in most of the States, and we do not hesitate to affirm that there is not one legalized prescriber, from the Atlantic to the Pacific, in Canada who would be so dense and ignorant as to suppose that a chloride of mercury was meant in the above prescription. The education of the pharmacist, like that of the dentist and the physician in Canada, starts in the right direction by a thorough preliminary examination almost equivalent to that of the Bachelor of Arts of our Universities. —"Dominion Dental Journal."

---

DISCOLORED TEETH.—All teeth that are discolored are without living pulps. Histologically they have no cells, pigment cells or any others. The dentin consists of formed matter which has been made by cells. It seems to me that the discoloration of teeth is caused probably by two conditions. The tooth structure, the dentin is open, it is traversed by the tubuli; now the tubuli may be filled with a substance which has a color, and which makes the tooth a different color from the rest of the teeth in the mouth, because the tubuli are filled with it. To bleach that tooth you must destroy the foreign colored substance which fills the tubuli.

Is there not another possibility for discoloration of the teeth, namely, that the dentin itself has been affected by something, so that it is of different composition, and the resulting compound of the dentin and some foreign substance has a color different from that of normal dentin? It stands to reason that one of these cases would bleach more easily than the other. I think very often you have the tubuli of the dentin filled with blood pigments which may be cause of discoloration, which can be bleached much more easily than in the second condition in which the dentin itself is chemically compounded with some substance which gives it a new composition and a different color. I do not understand the use of "pigment cells" in relation to the teeth in this connection at all. If the dentin is affected by a substance which combines with it, it may be less deeply affected, but it is a homogeneous substance which is affected, and not individual cells. Just the same as you may have an ivory billiard ball stained part way through or all the way through, but it has no stained cells, it has not pigment cells, but it is the staining of a definite substance, just as you have any other chemical substance which is changed in color by combination with another substance.—*Dr. F. B. Noyes, in Dental Review.*

---

SHALL THE DENTIST ADVERTISE ?—A correspondent in the *Items of Interest* says some good things in a recent article :

"The man who is seeking the road to wealth is misdirected when he chooses the dental profession. The average dentist lives well, spends freely dies poor and in debt."

"Keep out of the theatre programmes, for they are almost exclusively in the hands of the quacks, and for this reason the reputable man, feeling that there must be a dividing line between himself and the incompetent, refuses to advertise at all. I claim that if one city paper, of high standing, should make an effort to have a professional directory of medical and dental men, excluding the quacks and incompetents, society members exclusively of accepted standing, each with name and address, it would be a good thing for the paper, the public and the profession."

"Shall the man who has conducted himself in an unprofessional manner join the societies on an equal footing with the young man who is just entering practice and trying to conduct himself in strict accord with the code ethics ? Will he not also conclude to advertise and when he has secured a practice become a society member ? If we expect him to follow the straight and narrow path, the reward of improper conduct should not be the too easy admittance in the society."

We must not censure the custom of the profession before we were organized and recognized as a "profession." Many of our best men then advertized in a way that to-day is considered unethical. The times then were out of joint. We are professionally better now than then, and should behave better. —*Dominion Dental Journal.*

STUDENTS PRACTISING DENTISTRY.—There should be a severe example made in the colleges of the first student caught practising for free or reward on his own account. Such conduct in medicine or law is simply impossible, but in dentistry it has become a very general custom. Several cases have come under our own notice the last month, where second-year students had made full upper sets for five dollars "on the quiet," and for parties who where quite well able to patronize regular licentiates. In fact, the custom has become such a fad that the rooms of some of these students in boarding-houses are unlicensed offices on a small scale, and a system of toothing for patients has even extended on the sly to the infirmary patients.

It is generally this class of students who degrade the profession by cheap fees when they get into regular practice. It is a fact, too, that we have some very indigent young men in the ranks. Their poverty is no crime, but unfortunately they imagine it ought to justify them in breaches of law. The common thief may as consistently use the same argument.—"Dominion Dental Journal."

---

FASTIDIOUS OPERATING.—If there be any one thing which a dentist should cultivate, it is delicacy and lightness of touch. Some dentists whom we have known go at their work like a miner with a pick-axe. They are rough, harsh, and their hand, whether with the excavator, the plugger, or engaged in adjusting the various appliances of our art, is ever heavy. Their arms always rests burdensomely upon the patient's head. Their finger-nails are continually digging into tender tissues and there is a coarseness and clumsiness about their operations that marks an unpardonable heedlessness of the comfort of the patient. There are few things which so forcibly commend an operator to those under his care as tenderness and even daintiness, in regard to their sensibilities. The engine bur should be directed as if it were a sentient thing, and napkins should be used as though they were a spontaneous production.—Editor, in *Practitioner*.

